# Peripheral Syndecan-3 and Neurofilament Light Chain as Complementary Blood Biomarkers for Alzheimer’s Disease

**DOI:** 10.3390/ijms27031600

**Published:** 2026-02-06

**Authors:** Anett Hudák, Annamária Letoha, Tamás Letoha

**Affiliations:** 1 Pharmacoidea Ltd., H-6726 Szeged, Hungary; anett.hudak@pharmacoidea.eu; 2 Department of Medicine, Albert Szent-Györgyi Clinical Center, Faculty of Medicine, University of Szeged, H-6720 Szeged, Hungary; letohadr@gmail.com

**Keywords:** Alzheimer’s disease, neurofilament light chain, syndecan-3, peripheral blood mononuclear cells, biomarker, neuroinflammation, immune remodeling

## Abstract

Reliable and disease-specific blood biomarkers are critically needed for Alzheimer’s disease (AD), particularly in early stages when interventions are most effective. Although phosphorylated tau and neurofilament light chain (NfL) are widely used, their diagnostic specificity has been reported to decrease in elderly populations with multimorbidities. Syndecan-3 (SDC3), a heparan sulfate proteoglycan implicated in amyloid and tau aggregation, has recently emerged as a mechanistically relevant biomarker candidate. In this clinically realistic cohort study, we examined 46 participants, including 23 clinically diagnosed AD patients and 23 age-matched non-AD individuals with psychiatric and/or metabolic comorbidities. SDC3 expression was quantified in peripheral blood mononuclear cells (PBMCs), while soluble SDC3 and NfL were measured in plasma. Both PBMC-expressed and plasma SDC3 levels were elevated in AD compared with non-AD participants and showed a strong intercorrelation, whereas plasma NfL was likewise increased in AD. Individually, PBMC-SDC3, plasma SDC3, and NfL demonstrated moderate discriminatory performance. However, multivariable models integrating SDC3 (PBMC or plasma), NfL, and age achieved substantially improved discrimination (AUC > 0.8). SDC3 did not correlate with NfL, consistent with a biological signal distinct from neuroaxonal injury and reflective of peripheral immune–metabolic remodeling. Together, these findings identify SDC3 as a blood-based biomarker associated with systemic immune remodeling that complements established neuronal markers in a clinically realistic AD versus non-AD comparison. While exploratory, this study supports further investigation of SDC3 within integrated, multi-domain biomarker strategies in larger and independent cohorts.

## 1. Introduction

Alzheimer’s disease (AD) remains one of the most challenging neurodegenerative disorders to diagnose at a stage when disease-modifying interventions could be most effective [1,2,3]. Blood-based biomarkers such as phosphorylated tau (p-tau) and neurofilament light chain (NfL) have substantially advanced early detection efforts, yet both primarily capture downstream neurodegenerative processes: amyloid and tau pathology in the case of p-tau, and axonal injury for NfL [4,5,6,7]. Notably, NfL is broadly elevated across neurodegenerative conditions involving axonal damage, limiting its disease specificity for AD [8,9,10]. Likewise, in elderly populations with common comorbidities—including type 2 diabetes mellitus, chronic kidney disease, myocardial infarction, and stroke—plasma p-tau levels are frequently increased, leading to false positives in precisely those individuals at greatest risk of AD [11,12,13,14,15,16,17,18,19,20]. This lack of specificity in multimorbid clinical settings limits the utility of these biomarkers as stand-alone diagnostic tools.

Immune remodeling, by contrast, occurs early in AD pathogenesis and may provide a window into the disease before neuronal damage becomes irreversible. Syndecan-3 (SDC3)—a transmembrane heparan sulfate proteoglycan (HSPG) overexpressed in AD brains—is increasingly recognized as a mechanistically relevant factor in this process [21]. Experimental evidence shows that SDC3 promotes the aggregation and spreading of misfolded amyloid-β and tau and accelerates the formation of amyloid plaques and neurofibrillary tangles [22,23,24]. In the APP^SWE–Tau transgenic mouse model of AD, SDC3 is upregulated both in the central nervous system and in the periphery, while monocyte SDC3 expression positively correlates with cerebral amyloid burden [25].

Our pilot human study confirmed that peripheral blood mononuclear cell (PBMC)-expressed SDC3 is significantly elevated in patients with clinically diagnosed AD compared to non-AD individuals, while remaining low in multimorbid non-AD controls in whom plasma p-tau217 provided little diagnostic discrimination [26]. These findings position PBMC-SDC3 as a potential AD-associated biomarker and suggest that it may offer improved specificity in multimorbid patients compared with established neuronal markers. However, our prior study had two significant limitations. First, the non-AD control group was younger than the AD group, precluding a definitive assessment of AD specificity. Secondly, only the cell-associated form of SDC3 was measured, even though SDC3 also exists in a soluble form shed from cell surfaces under proteolytic and inflammatory conditions; whether this circulating pool carries the same disease-specific signal as leukocyte-expressed SDC3 is unknown.

To address these gaps, we analyzed both PBMC-expressed and plasma-soluble SDC3 in an age-matched cohort of patients with clinically diagnosed AD and cognitively unimpaired individuals with psychiatric and/or metabolic comorbidities. The multimorbid control population was deliberately selected to mirror real-world diagnostic conditions and to assess whether SDC3 can differentiate AD from clinically overlapping non-AD states, in which conventional biomarkers often lose specificity. Plasma NfL was included as a benchmark marker of neuroaxonal injury [27]. This study design enabled us to determine whether SDC3 distinguishes AD from non-neurodegenerative disorders despite overlapping comorbidity profiles, and to examine the relationship between the cellular and soluble forms of SDC3 and their association with NfL. Through this approach, we aimed to refine the peripheral immunological signature of AD and assess SDC3’s potential as a biologically distinct blood biomarker that captures disease-relevant systemic immune alterations complementary to established neuronal markers.

## 2. Results

### 2.1. Baseline Clinical and Immunological Features of the Study Cohorts

The study included 46 participants: 23 patients with clinically diagnosed AD and 23 cognitively unimpaired, age-matched non-AD controls with psychiatric and/or metabolic comorbidities. AD diagnoses were established by board-certified neurologists based on clinical assessment and neuroimaging, and coded according to ICD-10 criteria (G30.x; F00.x) [28]. As shown in Table 1, the two groups were demographically similar: age, sex distribution, BMI, and oxygen saturation did not differ significantly.

Comorbidities were frequent in both groups, reflecting the multimorbidity typical of elderly populations. All participants in the non-AD group had a diagnosis of hypertension (100%), compared with 13 individuals in the AD group (56.5%). Consistent with effective antihypertensive therapy, blood pressure (BP) values were within the normal or slightly elevated range and did not differ significantly between groups (Table 1). Cardiovascular disease was equally prevalent in both cohorts (78.3%). In contrast, psychiatric disorders (43.5% vs. 13.0%) and metabolic conditions, including diabetes mellitus (69.6% vs. 21.7%) and chronic kidney disease (17.4% vs. 8.7%), were more frequent in the non-AD group (Supplementary Table S1). This intentional inclusion of multimorbid controls, while enhancing real-world relevance for biomarker specificity testing, also introduces greater heterogeneity in systemic physiology.

Medication patterns reflected these comorbidity differences. Antihypertensive, cardiovascular, metabolic, and psychotropic drugs were widely used in both groups, whereas AD-specific therapies (acetylcholinesterase inhibitors and NMDA-receptor antagonists) were administered only in the AD group (Supplementary Table S2).

Routine hematological and biochemical parameters were generally comparable across cohorts (Supplementary Table S3). Complete blood counts and major organ function markers, including glucose, albumin, creatinine, urea, electrolytes, and liver enzymes, showed no significant differences, except for alkaline phosphatase (ALP) and gamma-glutamyl transferase (GGT), which were higher in non-AD participants (ALP: 106.65 ± 11.32 vs. 78.00 ± 4.95 U/L; *p* = 0.025; GGT: 56.13 ± 21.91 vs. 31.48 ± 9.14 U/L; *p* = 0.006). These elevations are consistent with the higher prevalence of metabolic and hepatic comorbidities in the non-AD cohort. Inflammatory and cardiac biomarkers, including CRP, IL-6, troponin T, and procalcitonin (PCT), did not differ between groups. (Given the exploratory nature of the study and the absence of adjustment for multiple comparisons, these differences are reported descriptively.)

Overall, the baseline characteristics indicate good demographic comparability between cohorts, with group differences arising mainly from comorbidity-driven factors. This provides a clinically realistic background against which the disease specificity of candidate biomarkers can be assessed.

### 2.2. Plasma NfL Levels Differentiate AD from Non-AD Participants

**Figure 1:**
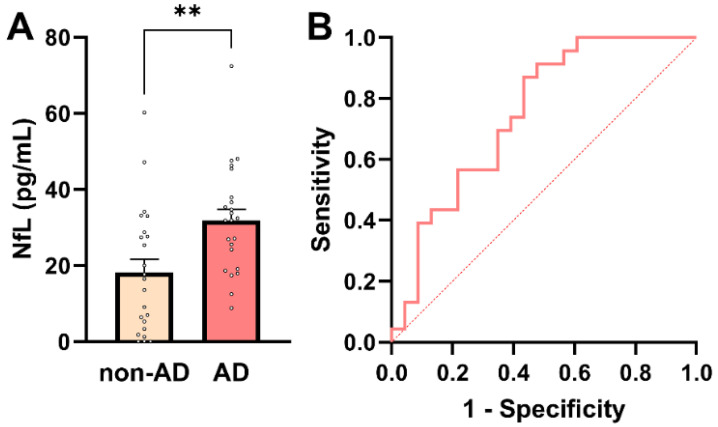
Plasma NfL concentrations in AD and non-AD participants. (**A**) Individual participant values with bars representing mean ± SEM. Plasma NfL levels were significantly higher in AD patients compared with non-AD controls (Mann–Whitney U-test, ** *p* = 0.004). (**B**) ROC curve for plasma NfL in distinguishing AD from non-AD participants. The dashed diagonal line indicates chance-level performance (AUC = 0.5).

Given its established role as a marker of neuroaxonal injury and its widespread use in AD diagnostics, plasma NfL was first evaluated in this cohort. Plasma NfL concentrations are known to exhibit right-skewed distributions in elderly and multimorbid populations, reflecting both biological variability and comorbidity-related influences. Consistent with this, assessment of distributional properties indicated deviation from normality in the non-AD group by the Shapiro–Wilk test (*p* = 0.0385), while other normality tests showed limited sensitivity at the present sample size (Supplementary Table S4). Given the small cohort size and the known distributional characteristics of plasma NfL, nonparametric statistical methods were applied for group comparisons. Plasma NfL concentrations were significantly higher in AD patients than in non-AD controls (median 31.9 pg/mL [IQR: 19.1–37.9] vs. 16.5 pg/mL [IQR: 3.35–28.8]; Mann–Whitney U = 396, *p* = 0.004; Figure 1A), confirming increased neuroaxonal injury in the AD group.

To further assess the diagnostic relevance of plasma NfL across the whole cohort (*n* = 46), association and predictive analyses were performed. Plasma NfL showed a significant positive association with AD status (point-biserial Pearson’s *r* = 0.413; 95% CI: 0.141–0.628; *p* = 0.004), indicating higher NfL concentrations in individuals with AD. In univariable logistic regression, plasma NfL emerged as a significant predictor of AD status (*β* = 0.0607 ± 0.0235; *p* = 0.0098), corresponding to an odds ratio of 1.063 (95% CI: 1.019–1.119). Thus, each 1 pg/mL increase in plasma NfL was associated with a 6.3% increase in the odds of AD. Receiver operating characteristic (ROC) analysis demonstrated acceptable discriminatory performance of plasma NfL for distinguishing AD from non-AD participants (AUC = 0.75; 95% CI: 0.61–0.89; *p* = 0.004; Figure 1B). A model-derived NfL concentration of approximately 24.7 pg/mL corresponded to a 50% predicted probability of AD. At this threshold, plasma NfL achieved a sensitivity of 70% and a specificity of 61%, with a positive predictive value (PPV) of 64% and a negative predictive value (NPV) of 67%. These metrics align with NfL’s known role as a sensitive, but not disease-specific, marker of neuroaxonal injury. Notably, plasma NfL levels showed a small but significant inverse correlation with PCT, a marker of systemic inflammation (*r* = −0.332; *p* = 0.03; Supplementary Figure S1). This unexpected association should be interpreted cautiously and is best considered as hypothesis-generating, given the modest sample size and the potential influence of comorbidity-related factors, renal clearance, or medication effects. Larger cohorts will be required to determine whether this relationship reflects a reproducible biological interaction or cohort-specific variability.

Taken together, these findings confirm plasma NfL as a strong marker of neuroaxonal injury, distinguishing AD from non-AD individuals in a multimorbid clinical population. At the same time, its only moderate diagnostic specificity highlights the need for additional biomarkers that reflect non-neuronal aspects of AD pathophysiology.

### 2.3. PBMC-Expressed SDC3 Is Elevated in AD Patients

**Figure 2:**
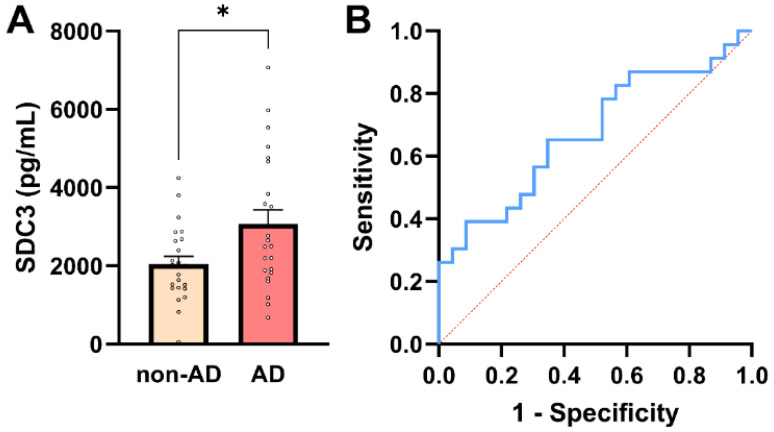
PBMC-expressed SDC3 concentrations in AD and non-AD participants. (**A**) PBMC-expressed SDC3 concentrations in AD patients (*n* = 23) and non-AD controls (*n* = 23). Bars show mean + SEM, with individual participant values overlaid. SDC3 levels were significantly higher in AD compared with non-AD participants (* *p* = 0.016, unpaired two-tailed *t*-test). (**B**) ROC curve of PBMC-expressed SDC3 for discriminating AD from non-AD individuals. The dashed diagonal line represents chance-level classification (AUC = 0.68; 95% CI: 0.52–0.83).

SDC3 expression was quantified in peripheral PBMCs from all study participants. To maximize assay sensitivity while maintaining linearity of signal detection, 3 × 10^5^ PBMCs per sample were used for SDC3 quantification by ELISA. Assessment of distributional properties confirmed approximate normality of PBMC-expressed SDC3 values in both groups (all normality tests *p* > 0.05; Supplementary Table S5), justifying the use of parametric statistical methods for group comparisons. As shown in Figure 2A, PBMC-expressed SDC3 concentrations were significantly higher in AD patients than in non-AD controls (mean ± SEM: 3069.24 ± 359.1 pg/mL vs. 2040.19 ± 201.52 pg/mL, respectively; unpaired two-tailed *t*-test, *p* = 0.016). Individual data points revealed moderate inter-individual variability, but an apparent upward shift in SDC3 expression in the AD group.

Across the whole cohort (*n* = 46), PBMC-expressed SDC3 showed a moderate positive association with AD status (Pearson *r* = 0.353, *p* = 0.016). In univariable logistic regression, PBMC-SDC3 emerged as a statistically significant predictor of AD (*β* = 0.0006 ± 0.0003; *p* = 0.028). The corresponding odds ratio was modest (OR = 1.001; 95% CI: 1.000–1.001), reflecting the continuous nature of the biomarker and the fine-unit scale of measurement, rather than a weak biological effect.

ROC analysis demonstrated intermediate discriminatory performance of PBMC-expressed SDC3 for distinguishing AD from non-AD participants (AUC = 0.68; 95% CI: 0.52–0.83; *p* = 0.042; Figure 2B). A model-derived PBMC-SDC3 concentration of approximately 2485 pg/mL corresponded to a predicted probability of 50% for AD. At this threshold, sensitivity was 56.5% and specificity 69.6%, with a positive predictive value (PPV) of 65.0% and a negative predictive value (NPV) of 61.5%, consistent with moderate stand-alone discriminatory accuracy.

Beyond disease status, PBMC-expressed SDC3 exhibited significant inverse correlations with multiple systemic laboratory parameters, including PCT (*r* = −0.439, 95% CI: −0.654 to −0.160; *p* = 0.0032), alkaline phosphatase (ALP; *r* = −0.416, 95% CI: −0.630 to −0.143; *p* = 0.0040), C-reactive protein (CRP; *r* = −0.331, 95% CI: −0.572 to −0.037; *p* = 0.0283), and urea (*r* = −0.323, 95% CI: −0.567 to −0.027; *p* = 0.0285). The consistency of these inverse associations suggests that higher PBMC-SDC3 expression is linked to a systemic milieu characterized by lower inflammatory and metabolic stress, supporting the interpretation of SDC3 as a marker of chronic immune remodeling rather than acute inflammation. In contrast, PBMC-expressed SDC3 did not show a significant association with plasma NfL levels (*r* = 0.244; 95% CI: −0.049 to 0.509; *p* = 0.102; Supplementary Table S6), further reinforcing the notion that SDC3 primarily reflects peripheral immune and metabolic processes distinct from neuroaxonal injury.

### 2.4. Plasma SDC3 Concentrations in AD and Non-AD Groups

**Figure 3:**
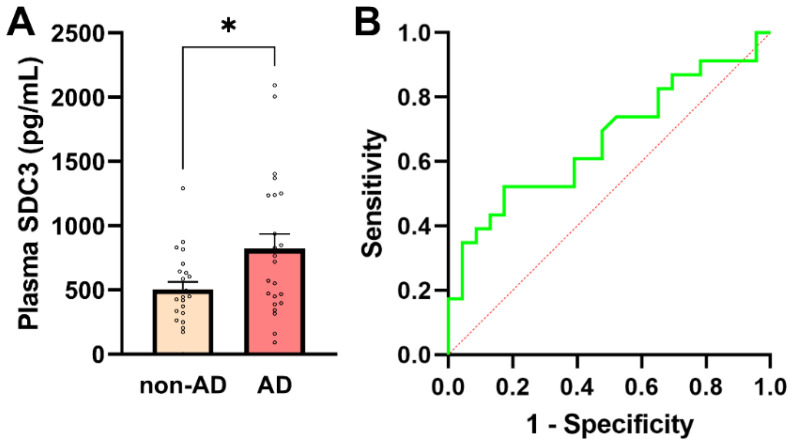
Plasma SDC3 concentrations in AD and non-AD participants. (**A**) Plasma SDC3 levels were significantly higher in AD compared with non-AD participants (unpaired two-tailed *t*-test, * *p* = 0.019). Bars represent mean + SEM, with individual data points overlaid. (**B**) ROC curve for plasma SDC3 in discriminating AD from non-AD participants. The dashed diagonal line denotes chance-level classification (AUC = 0.67; 95% CI: 0.508–0.825). The *x*-axis represents the false-positive rate (1-specificity) and the *y*-axis the true-positive rate (sensitivity).

In addition to assessing cellular SDC3 expression, we quantified plasma concentrations of soluble SDC3, which represent the proteoglycan’s shed extracellular form. Plasma SDC3 concentrations showed normality in both groups (all normality tests *p* > 0.05), justifying the use of parametric statistical testing (Supplementary Table S7). As shown in Figure 3A, plasma SDC3 levels were significantly higher in AD patients (821.65 ± 113.92 pg/mL) than in non-AD individuals (505.01 ± 58.42 pg/mL; *p* = 0.019, unpaired two-tailed *t*-test). A strong and highly significant correlation was observed between plasma and PBMC-expressed SDC3 levels (*r* = 0.921; *p* < 0.0001). This robust association is consistent with shared biological regulation, including coordinated cellular expression and shedding of SDC3, rather than methodological dependency, as the two measurements were obtained from independent biological matrices.

Plasma SDC3 also showed a modest but statistically significant positive correlation with AD status (*r* = 0.349; 95% CI: 0.066–0.581; *p* = 0.017), indicating that circulating SDC3 levels tend to be higher in individuals with AD. Logistic regression analysis further supported this association, revealing a statistically significant positive slope (*β* = 0.00191 ± 0.00088; *p* = 0.0304) and an odds ratio of 1.002 (95% CI: 1.000–1.004). The 50% classification threshold was reached at a plasma SDC3 concentration of approximately 637.5 pg/mL. At this cutoff, plasma SDC3 correctly identified 52.2% of AD patients (sensitivity) and 73.9% of non-AD participants (specificity), with a PPV of 66.7% and an NPV of 60.7%. Model performance was modest (AUC = 0.667; 95% CI: 0.508–0.825; *p* = 0.0532), consistent with these diagnostic metrics (Figure 3B).

Plasma SDC3 concentrations showed significant negative correlations with multiple systemic laboratory parameters. The strongest negative associations were observed with ALP (*r* = −0.425, *p* = 0.0032), PCT (*r* = −0.392, *p* = 0.0094), and CRP (*r* = −0.375, *p* = 0.0103), consistent with an anti-inflammatory or immunomodulatory profile (Supplementary Table S8). In contrast, plasma SDC3 levels did not significantly correlate with NfL (*r* = 0.244, *p* = 0.102), suggesting that SDC3 reflects peripheral inflammatory processes more than neurodegeneration-associated axonal damage.

### 2.5. Blood-Based SDC3 in the Context of Antihypertensive Therapy

**Figure 4:**
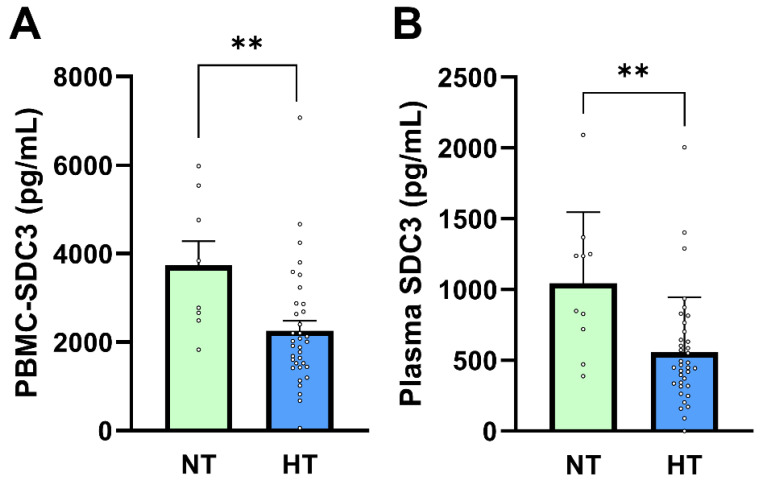
PBMC-SDC3 and plasma SDC3 concentrations in NT and HT participants. (**A**) PBMC-SDC3 and (**B**) plasma SDC3 levels were significantly lower in HT (*n* = 36) compared with NT (*n* = 10) participants (** *p* = 0.007 and ** *p* = 0.003, respectively). Bars represent mean + SEM for descriptive visualization, with individual participant values overlaid. Group comparisons were performed using nonparametric Mann–Whitney U-tests. ** *p* < 0.01.

In our previous study, PBMC-expressed SDC3 was inversely associated with diagnosed hypertension, independent of measured BP values [26]. In the present cohort, systolic and diastolic BP did not differ significantly between AD and non-AD participants, consistent with effective long-term antihypertensive treatment. Given this context, hypertension status in this study primarily reflects chronic exposure to antihypertensive therapy rather than current hemodynamic load. We therefore examined whether PBMC-expressed and plasma SDC3 levels were associated with hypertension status and antihypertensive medication use. PBMC-expressed SDC3 concentrations were significantly lower in hypertensive (HT) compared with normotensive (NT) participants (2010 ± 223 pg/mL vs. 3732 ± 545 pg/mL; Mann–Whitney U-test, *p* = 0.0073; Figure 4A). Logistic regression analysis supported this association (*β* = −0.00069 ± 0.00027; *p* = 0.011; OR = 0.9993; 95% CI: 0.9987–0.9998). Plasma SDC3 showed a similar pattern, with lower concentrations observed in HT individuals (1044 ± 158.9 pg/mL in NT vs. 557.5 ± 64.96 pg/mL in HT participants; Mann–Whitney U-test, *p* = 0.0073; Figure 4B). Plasma SDC3 correlated negatively with hypertension status (*r* = −0.443; 95% CI: −0.650 to −0.176; *p* = 0.002) and was likewise a significant predictor in logistic regression (*β* = −0.00228 ± 0.00090; *p* = 0.0108; OR = 0.9977; 95% CI: 0.9957–0.9993).

ROC analysis indicated good discrimination of hypertension status by plasma SDC3 within this cohort (AUC = 0.803; 95% CI: 0.654–0.952; *p* = 0.004). Given the small size of the normotensive subgroup (*n* = 10), this performance should be interpreted as exploratory, supporting a strong association between plasma SDC3 and exposure to antihypertensive treatment rather than establishing a diagnostic classifier. AD status itself was moderately and inversely associated with hypertension (*r* = −0.527; *p* = 0.0002), reflecting the higher prevalence and intensity of long-term antihypertensive therapy in the non-AD group. This pattern is consistent with epidemiological observations linking antihypertensive treatment to reduced AD risk, although causality cannot be inferred from the present cross-sectional design [29,30,31,32,33,34].

To further explore treatment-related effects, antihypertensive medication classes were examined (Table 2). Use of ACEi showed a significant inverse association with AD status (*φ* = −0.309; *p* = 0.037), whereas no other antihypertensive drug class demonstrated a significant relationship. In addition, the total number of antihypertensive medication classes used was significantly lower in AD patients than in non-AD participants (Pearson *r* = −0.379, *p* = 0.009; Spearman *ρ* = −0.383, *p* = 0.0085), indicating reduced overall antihypertensive treatment intensity in the AD group.

**Figure 5:**
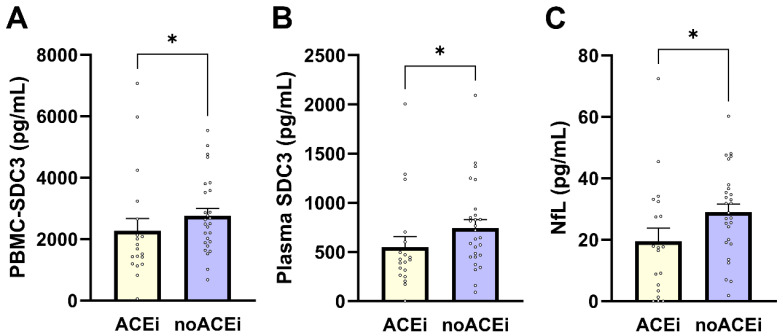
Effects of ACE inhibitor therapy on PBMC-expressed SDC3, plasma SDC3, and plasma NfL levels. Bars represent mean ± SEM for descriptive visualization, with individual participant values overlaid. (**A**) PBMC-expressed SDC3, (**B**) plasma SDC3, and (**C**) plasma NfL concentrations in ACEi users and non-users (noACEi). Group comparisons were performed using nonparametric Mann–Whitney U-tests. * *p* < 0.05.

PBMC-expressed SDC3 levels were significantly lower in individuals receiving ACE inhibitor (ACEi) therapy compared with non-users (median 1683 vs. 2501 pg/mL; Mann–Whitney U = 350.0; *p* = 0.038; Figure 5A). PBMC-SDC3 also declined with increasing antihypertensive regimen complexity, as reflected by a significant negative correlation with the number of antihypertensive drug classes used (Spearman *ρ* = −0.368; *p* = 0.0118). Plasma SDC3 showed parallel treatment-related patterns: ACEi users exhibited lower circulating SDC3 concentrations than non-users (mean 548.6 vs. 744.0 pg/mL; *p* = 0.023; Figure 5B), and plasma SDC3 correlated negatively with antihypertensive treatment intensity (Spearman *ρ* = −0.35; *p* = 0.018). Together, these findings indicate that renin–angiotensin system–related pharmacological exposure is consistently associated with reduced SDC3 expression in both cellular and soluble compartments. Finally, we assessed whether ACE inhibitor therapy was also associated with plasma NfL. Plasma NfL concentrations were significantly lower in ACEi users compared with non-users (median 17.41 vs. 28.75 pg/mL; Mann–Whitney U = 154.0; *p* = 0.022; Figure 5C), corresponding to an approximate 11 pg/mL difference. This association was supported by nonparametric analysis (Spearman *ρ* = −0.341; *p* = 0.020), with point-biserial Pearson correlation showing a similar trend (*r* = −0.282; *p* = 0.057).

Taken together, these results demonstrate that antihypertensive treatment, particularly ACEi-exposure and overall treatment intensity, is consistently associated with lower PBMC-expressed SDC3, plasma SDC3, and plasma NfL levels. These findings underscore the importance of accounting for antihypertensive medication use when interpreting immune- and neurodegeneration-associated blood biomarkers in multimorbid older adults and support further investigation of treatment–biomarker interactions in larger, longitudinal cohorts.

### 2.6. Integrated Biomarker Models Enhance Cohort-Level Discrimination

**Figure 6:**
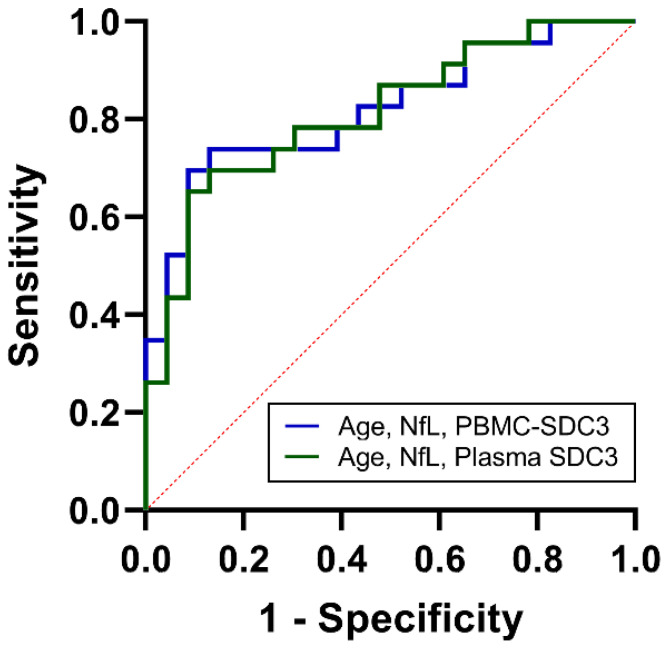
ROC analysis of combined biomarkers for cohort-level discrimination between AD and non-AD participants. ROC curves for multivariable logistic regression models integrating age, NfL, and PBMC-expressed SDC3 (blue) or plasma SDC3 (green). Both combined models demonstrated improved discrimination compared with individual biomarkers, with AUC values of 0.82 (PBMC model) and 0.81 (plasma model). The *x*-axis represents the false positive rate (1-specificity), and the *y*-axis represents the true positive rate (sensitivity).

As noted above, PBMC-expressed SDC3 showed intermediate discriminative power (AUC = 0.68, 95% CI: 0.52–0.83; *p* = 0.042). Plasma yielded similar classification accuracy (AUC = 0.67, 95% CI: 0.51–0.83; *p* = 0.053), suggesting that soluble SDC3 reflects overlapping but distinct aspects of systemic immune remodeling. Plasma NfL, in contrast, provided stronger discrimination (AUC = 0.75, 95% CI: 0.61–0.89; *p* = 0.004), in line with its established role as a marker of neuroaxonal injury. Age alone showed weak predictive value (AUC = 0.57, 95% CI: 0.40–0.74). To determine whether combined biomarker assessment could improve classification, we constructed multivariable logistic regression models. The PBMC-based model, incorporating PBMC-expressed SDC3, plasma NfL, and age, achieved an AUC of 0.82 (95% CI: 0.71–0.94; *p* < 0.001), with a Nagelkerke R^2^ of 0.41 (Figure 6). The plasma-based model, integrating plasma SDC3, plasma NfL, and age, performed comparably, with an AUC of 0.81 (95% CI: 0.69–0.93; *p* < 0.001) and a Nagelkerke R^2^ of 0.39. Both integrated models substantially outperformed individual biomarkers, indicating that SDC3—whether measured in PBMCs or plasma—adds diagnostic value when combined with markers of neuroaxonal injury and demographic risk. These findings demonstrate that while SDC3 and NfL individually offer intermediate discriminatory accuracy, their integration with age markedly enhances discrimination. The near-identical performance of plasma- and PBMC-based panels highlights the potential clinical utility of measuring soluble SDC3 as a simpler alternative to cell-based assays.

## 3. Discussion

Due to its high prevalence and devastating disease course, along with the persistent difficulty of making an early and accurate diagnosis in clinically heterogeneous patient populations, AD remains one of the most significant challenges in modern neurology [35]. While amyloid- and tau-based biomarkers have transformed the diagnostic landscape, their use in real-world multimorbid populations is limited [11]. Plasma phosphorylated tau, for example, is frequently elevated in common chronic conditions such as diabetes, kidney disease, myocardial infarction, and stroke, while NfL is increased across a broad range of neurodegenerative disorders, limiting its specificity [8,9,10,13,14,16,17,18,19,20].

Against this background, our study identifies SDC3, a HSPG involved in immune regulation and cell signaling, as a novel blood-based biomarker candidate. Both PBMC-expressed and plasma SDC3 concentrations were elevated in AD compared with non-AD individuals, and the two measures correlated closely. On their own, SDC3 levels showed only modest discriminatory power (AUC ~ 0.67–0.68). However, when combined with NfL and age, cohort-level discrimination improved substantially (AUC > 0.8), comparable to the best-performing plasma biomarker panels reported to date. The nearly identical performance of PBMC- and plasma-based models suggests that soluble SDC3 could serve as a clinically practical alternative to cell-based assays, pending validation in independent cohorts.

The biological associations of SDC3 also provide important insights. PBMC-expressed SDC3 correlated inversely with systemic inflammatory and metabolic markers, including PCT, CRP, ALP, and urea. Plasma SDC3 showed similar patterns, supporting the idea that SDC3 reflects peripheral immune and metabolic processes rather than neuroaxonal damage. Importantly, SDC3 did not correlate with NfL, reinforcing its role as a marker of systemic immune biology rather than neurodegeneration per se.

A particularly striking finding was the consistent reduction in SDC3 in participants with hypertension, despite comparable blood pressure values between hypertensive and normotensive groups. This likely reflects the effects of long-term antihypertensive therapy rather than vascular load itself. Logistic regression confirmed good cohort-level discrimination for hypertension status, and AD status correlated negatively with hypertension, consistent with epidemiological evidence that antihypertensive medications—particularly ACE inhibitors, angiotensin receptor blockers, calcium channel blockers, and beta-blockers—are associated with reduced AD risk [29,30,31,32,33,34]. However, the present cross-sectional design does not allow causal inference, and observed associations may reflect treatment exposure patterns and comorbidity structure. Future studies with stratification by antihypertensive class are needed to confirm therapy-specific effects.

When compared with other immune- and glia-derived biomarkers, SDC3 adds a complementary dimension. Soluble TREM2 and YKL-40 reflect microglial and astrocytic activation, while plasma GFAP is a sensitive marker of reactive astrocytosis [36,37,38,39]. These molecules capture central neuroimmune responses [40]. In contrast, SDC3 represents peripheral immune remodeling and captures a systemic immune–metabolic dimension of AD-related biology, thereby broadening the biomarker spectrum to processes that have long been implicated in AD but remain underexplored. The modest performance of SDC3 alone should therefore not be viewed as a limitation. Like TREM2, YKL-40, or GFAP, its strength lies in complementing other markers. In integrated models, SDC3 improved discrimination beyond NfL and age, underscoring the value of multi-domain biomarker strategies that incorporate both central and peripheral signals.

The inverse correlation between NfL and PCT observed in this study further underscores the importance of accounting for systemic inflammation when interpreting biomarkers. This suggests that variability in NfL levels across patients may partly reflect inflammatory status, complicating its role as a stand-alone diagnostic marker. However, this finding should be considered hypothesis-generating and requires replication in larger, independent cohorts, given that prior studies typically report elevated NfL in systemic inflammatory states.

The detection of SDC3 in plasma is especially encouraging for translation. Plasma assays are less invasive and easier to standardize than PBMC-based assays, making them more suitable for large-scale clinical application, while PBMC measurements remain useful for mechanistic research.

This study has several limitations. This study has several limitations. A key limitation is that although AD diagnoses were established according to routine clinical practice and internationally accepted criteria, the biomarker measurements supporting these diagnoses (e.g., CSF- or PET-based amyloid or tau assessments) were not consistently available in the clinical records for research-level analysis. This limitation reflects restricted access to underlying biomarker data rather than uncertainty regarding the clinical diagnosis itself. Accordingly, future studies specifically designed for biomarker validation should include cohorts in which biomarker data are systematically available to enable biological stratification alongside clinical diagnosis.

The modest cohort size and cross-sectional design also limit causal inference and increase susceptibility to model overfitting. In addition, the inclusion of psychiatric and metabolic comorbidities in the non-AD control group, while intentionally reflecting real-world multimorbidity, may introduce confounding. This design sacrifices maximal biological contrast in favor of clinical realism, thereby strengthening the translational relevance of the findings.

PBMC-associated SDC3 measurements reflect total cell-associated protein abundance and do not account for leukocyte subpopulation differences. Given the modest sample size, multivariable logistic regression models should be considered exploratory, as they were not subjected to internal cross-validation or penalized regression. Although the observed AUC values were robust, these findings require validation in larger, independent, and longitudinal cohorts to clarify temporal relationships between SDC3 elevation, cognitive decline, and treatment exposure, and to determine whether SDC3 alterations precede, coincide with, or follow immune remodeling relative to neuroaxonal injury.

In summary, SDC3 emerges as a novel immune-related biomarker associated with AD that reflects systemic biology rather than central neurodegeneration alone. Both cellular and plasma SDC3 levels were elevated in AD, inversely associated with systemic inflammation, and reduced in individuals receiving antihypertensive therapy. While its stand-alone discriminatory performance was modest, SDC3 enhanced cohort-level discrimination in multi-domain biomarker panels. These findings highlight SDC3 as a promising candidate for integrated biomarker strategies and emphasize the need for further studies to clarify its utility for clinically robust discrimination, risk stratification, and therapeutic monitoring of AD.

## 4. Materials and Methods

### 4.1. Study Population and Data Collection

This study was approved by the Regional and Institutional Review Board of the University of Szeged (Approval Number: 99/2022-SZTE RKEB, approval date: 29 August 2022). The protocol was reviewed from both ethical and scientific perspectives in accordance with Decree 23/2002 of the Hungarian Ministry of Health and Government Decree 235/2009 (X.20.) and was conducted in compliance with the Declaration of Helsinki. All participants provided written informed consent before enrollment. Data were collected and processed in anonymized form to ensure participant confidentiality.

The sample size (*n* = 46; 23 AD, 23 non-AD) was determined based on feasibility for this exploratory study. Accordingly, all statistical analyses were interpreted as hypothesis-generating, with emphasis placed on observed effect sizes and consistency of findings rather than confirmatory power considerations.

Clinical, neuropsychological, and comorbidity information was extracted from hospital records. Demographic and diagnostic data were collected with a specific focus on AD. AD diagnoses were established by board-certified neurologists based on clinical presentation, longitudinal medical history, and structural neuroimaging, and were coded according to ICD-10 criteria (G30.x; F00.x) [28,41,42]. Standardized quantitative neuropsychological test scores (e.g., MMSE, MoCA, CDR) were routinely used as part of the clinical diagnostic process; however, these scores were not uniformly available in a structured, research-grade format in the hospital records due to the retrospective, real-world nature of the cohort, and therefore could not be incorporated into the present research analyses.

In addition to AD diagnosis, key demographic variables such as age, sex, and BMI were recorded. Comorbidity data were also systematically collected, including the presence of hypertension, cardiovascular diseases, chronic lung diseases, psychiatric or mental health disorders, and diabetes mellitus, among others.

Peripheral blood samples were collected and subjected to standard laboratory analyses, including complete blood counts, inflammatory markers, hepatic and cardiac enzymes, and selected AD-related biomarkers.

To ensure data integrity, all clinical and laboratory entries were independently reviewed and cross-validated by two researchers. This dual-check process minimized potential biases and supported the robustness of subsequent statistical analyses.

### 4.2. Plasma Preparation and Storage

Plasma preparation was performed essentially as previously described [26]. Briefly, 5 mL of venous blood was collected into sterile EDTA-coated tubes and processed immediately to prevent coagulation. Samples were centrifuged at 2000× *g* for 10 min at 4 °C to separate plasma from cellular components. The plasma fraction was carefully aspirated and transferred to sterile microcentrifuge tubes to minimize contamination and hemolysis. Aliquots of 50 µL were prepared and stored at −20 °C until analysis and were subjected to a single freeze–thaw cycle only at the time of biomarker measurement; no repetitive freeze–thaw cycles occurred.

### 4.3. Isolation of PBMCs from Whole Blood

Isolation of PBMCs was performed essentially as described previously [26]. Briefly, 6.0 mL of whole blood was collected into EDTA-coated tubes and layered onto an equal volume of HISTOPAQUE^®^-1077 (cat. no. H8889, Sigma-Aldrich) in 15 mL conical centrifuge tubes after equilibration to room temperature. Samples were centrifuged at 4000× *g* for 40 min at room temperature to allow density-based separation of mononuclear cells.

Following centrifugation, the plasma fraction was carefully aspirated, and the PBMC-containing buffy coat layer was transferred into a clean conical tube. Cells were washed by gentle resuspension in 1 mL phosphate-buffered saline (PBS) and centrifuged at 300× *g* for 10 min. The supernatant was discarded, and cells were fixed by incubation with 500 µL of 4% paraformaldehyde (PFA, cat. no. J61899.AK, Thermo Fisher Scientific, Waltham, MA, USA) at room temperature for 10 min. After fixation, cells were washed once with PBS, centrifuged again at 300× *g* for 10 min, resuspended in 1 mL PBS, and stored at 4 °C until further analysis.

### 4.4. Human Plasma NfL ELISA Measurements

Human plasma NfL levels were quantified using a sandwich enzyme-linked immunosorbent assay (ELISA) with the Human NfL ELISA Kit (cat. no.: ABIN6968865; antibodies-online Inc., Pottstown, PA, USA). The 96-well microplate supplied with the kit was pre-coated with a capture antibody specific to human NfL.

Plasma samples were thawed on ice, centrifuged to remove particulates, and diluted according to the manufacturer’s recommendation (minimum 1:2 dilution in Sample Dilution Buffer) to ensure that measured concentrations fell within the validated detection range of the assay (15.625–1000 pg/mL). For each assay plate, 100 µL of NfL standards, diluted samples, and Sample Dilution Buffer (blank) were added to individual wells, and the plate was incubated at 37 °C for 90 min. After incubation, wells were washed twice with Wash Buffer. Next, 100 µL of the biotin-labeled anti-NfL detection antibody—prepared freshly at a 1:100 dilution in Antibody Dilution Buffer—was added to all wells. Plates were incubated at 37 °C for 60 min, then washed three times. Subsequently, 100 µL of HRP-streptavidin conjugate (SABC), also diluted 1:100 in SABC Dilution Buffer, was added to each well and incubated at 37 °C for 30 min. Wells were then washed five times to remove unbound conjugate.

For color development, 90 µL of TMB substrate was added to each well and incubated at 37 °C in the dark for 10–20 min, until a clear color gradient appeared in the standard curve wells. The enzymatic reaction was stopped by adding 50 µL of Stop Solution, resulting in a yellow color. Absorbance was measured immediately at 450 nm using a BioTek Cytation 3 multimode microplate reader (BioTek Instruments, Winooski, VT, USA).

All standards and samples were assayed in duplicate, and reported concentrations represent the mean of replicate wells. No signal saturation or out-of-range values were observed, and all sample measurements fell within the linear dynamic range of the standard curve. Inter-replicate variability consistently remained below 20% (except for one sample near the assay detection limit), confirming assay reliability. Replicate data are summarized in Supplementary Table S9.

### 4.5. ELISA Measurements for SDC3 Quantification in PBMC Samples and Plasma

Quantification of SDC3 in both peripheral blood mononuclear cells (PBMCs) and plasma was performed using a commercial sandwich enzyme-linked immunosorbent assay (ELISA) (Human SDC3 ELISA Kit, cat. no. ABIN4884706; antibodies-online Inc.), according to the manufacturer’s instructions. The assay is specific for human SDC3 and validated for plasma samples, with a reported detection range of 0.061–15 ng/mL.

PBMC-associated SDC3 was quantified essentially as described previously [26], with an increased cell input to enhance assay sensitivity. PBMCs were isolated from whole blood by density-gradient centrifugation using HISTOPAQUE^®^-1077 (cat. no. H8889; Sigma-Aldrich, St. Louis, MO, USA) and washed twice with phosphate-buffered saline (PBS; pH 7.4). Equal numbers of PBMCs (3 × 10^5^ cells per well) were used for all samples to ensure consistency across measurements and to avoid signal saturation.

To minimize inter-assay variability and batch effects, PBMC-associated and plasma SDC3 samples (*n* = 46 each) were analyzed in parallel within the same ELISA run. For PBMC-associated measurements, cells were added directly to pre-coated 96-well microplates together with SDC3 standards (100 µL per well) and incubated for 2.5 h at room temperature. Frozen plasma samples were thawed on ice, centrifuged to remove particulate matter, and diluted 1:2 in assay diluent as recommended by the manufacturer. Standards and diluted plasma samples (100 µL per well) were added to the same pre-coated microplates and incubated for 2.5 h at room temperature.

After three washing steps, wells were incubated with 100 µL of biotin-conjugated anti-SDC3 detection antibody for 1 h at room temperature, followed by additional washing and incubation with streptavidin–horseradish peroxidase (HRP) conjugate for 45 min. After final washing, 100 µL of TMB substrate solution was added to each well, and color development proceeded for 30 min at room temperature in the dark. The enzymatic reaction was terminated by the addition of 50 µL stop solution, and absorbance was measured immediately at 450 nm using a BioTek Cytation 3 multimode microplate reader (BioTek Instruments; Winooski, Vermont, United States). All quantifiable measurements fell within the validated dynamic range of the assay.

### 4.6. Statistical Analyses

Data are presented as mean ± standard error of the mean (SEM). Group comparisons were performed using unpaired two-tailed *t*-tests or nonparametric Mann–Whitney *U*-tests, as appropriate, with *p* < 0.05 considered statistically significant. All statistical analyses and visualizations were conducted using IBM^®^ SPSS^®^ Statistics version 26 and GraphPad Prism version 9.

Given the exploratory nature of the study, all statistical analyses were interpreted as hypothesis-generating, with emphasis placed on observed effect sizes, confidence intervals, and internal consistency of findings rather than confirmatory inference. No formal adjustment for multiple comparisons was applied, and reported *p*-values are therefore interpreted descriptively.

Pearson or Spearman correlation analyses were used, as appropriate, to assess associations between SDC3, NfL, and systemic inflammatory, hepatic, and hematologic markers.

Receiver operating characteristic (ROC) analyses were performed to evaluate the cohort-level discriminatory performance of individual biomarkers. Binary logistic regression was used to model AD status (1 = AD, 0 = non-AD) as the dependent variable, with SDC3, NfL, and age included as predictors. The discriminatory ability of univariable and multivariable models was compared using the area under the ROC curve (AUC).

Sensitivity and specificity values are reported descriptively at model-derived probability thresholds and were not subjected to resampling-based confidence interval estimation due to the exploratory study design.

Normality of continuous variables was assessed using the Shapiro–Wilk test. For variables that deviated from normality, nonparametric tests were applied as appropriate.

## Figures and Tables

**Table 1:** Demographic comparison of the non-AD vs. the AD groups (values are mean ± SEM).

Parameter	Non-AD (*n* = 23)	AD (*n* = 23)	*p*-Value
Age (years)	78.48 ± 1.64	80.61 ± 1.35	0.32
Sex (% male)	39.1%	39.1%	1
BMI (kg/m^2^)	24.53 ± 1.57	23.15 ± 0.71	0.40
SpO_2_ (%)	96.4 ± 0.5	96.4 ± 0.5	0.97
Systolic BP (mmHg)	144.4 ± 5.2	130.5 ± 4.6	0.053
Diastolic BP (mmHg)	75.7 ± 3.3	76.3 ± 2.7	0.28

**Table 2:** Distribution of antihypertensive medication classes in study participants.

Medication Class	AD (*n* = 23)	Non-AD (*n* = 23)
ACE inhibitors (ACEi)	6	13
Angiotensin II receptor blockers (ARB)	3	8
Calcium channel blockers (CCB)	3	8
β-blockers (BB)	11	13
Thiazide/thiazide-like diuretics	3	6
Loop diuretics	3	5
Mineralocorticoid receptor antagonists (MRA)	0	1
Centrally acting antihypertensives	2	0
α1-adrenergic blockers	1	3
Total antihypertensive classes used (sum)	32	57
Any antihypertensive use (≥1 drug class)	17	21

Distribution of antihypertensive medication classes in AD and non-AD participants. Counts reflect the number of individuals using each class. AD participants used fewer antihypertensive classes overall, consistent with lower treatment intensity.

## Data Availability

Data are contained within the article or Supplementary Materials.

## Supplementary Materials

The following supporting information can be downloaded at https://www.mdpi.com/article/10.3390/ijms27031600/s1.
