# Supplementary material for: Peripheral Syndecan-3 and Neurofilament Light Chain as Complementary Blood Biomarkers for Alzheimer’s Disease
Source: Int J Mol Sci. 2026 Feb 6;27(3):1600. doi: 10.3390/ijms27031600 (PMC12898001; doi:10.3390/ijms27031600)
Supplement: Supplementary file 1 [file ijms-27-01600-s001.zip › ijms-4094755-supplementary.pdf]

Int. J. Mol. Sci. **2026**, *27*(3) https://doi.org/10.3390/xxxxx

Supplementary Materials

Peripheral Syndecan-3 and Neurofilament Light Chain as Complementary Blood Biomarkers for Alzheimer's Disease

Anett Hudák [1], Annamária Letoha [2], and Tamás Letoha [1,*]

Supplementary Table 1. Prevalence of comorbid conditions in non-AD and AD groups.

Comorbidity	non-AD Group (n = 23)	AD Group (n = 23)
Hypertension	23 (100.0%)	13 (56.5%)
Cardiovascular disease	18 (78.3%)	18 (78.3%)
Diabetes mellitus (any)	16 (69.6%)	5 (21.7%)
Insulin therapy (IDDM)	3 (13.6%)	2 (8.7%)
Chronic lung disease	4 (17.4%)	4 (17.4%)
Renal insufficiency	4 (17.4%)	2 (8.7%)
Autoimmune disorders	2 (8.7%)	3 (13.0%)
Obesity	5 (22.7%)	2 (8.7%)
Depression	8 (34.8%)	2 (8.7%)
Bipolar disorder	1 (4.3%)	1 (4.3%)
Schizophrenia	1 (4.3%)	1 (4.3%)
Parkinson's disease	3 (13.0%)	0 (0%)
Thyroid disorders	4 (17.4%)	3 (13.0%)
Gynecological conditions	4 (17.4%)	1 (4.3%)

The table summarizes the frequency and percentage of common comorbidities in the non-AD (n = 23) and the AD group (n = 23), including cardiovascular, metabolic, autoimmune, pulmonary, and neuropsychiatric conditions. Comorbidities were identified based on clinical history, medication records, and physician diagnosis. "Neuropsychiatric (other)" includes major neurological or psychiatric disorders other than Alzheimer's disease, specifically schizophrenia and Parkinson's disease. "Insulin therapy" refers to individuals receiving any form of insulin treatment (insulin-dependent diabetes mellitus – i.e., IDDM). Gynecological conditions were only assessed in female participants.

Supplementary Table 2. Medication profiles of AD and non-AD participants

Medication Class	Examples of Agents Used	AD	non-AD
ACE inhibitors	Perindopril, ramipril, enalapril, captopril	10 (43.5%)	14 (60.9%)
Angiotensin II receptor blockers (ARBs)	Valsartan, telmisartan, irbesartan	8 (34.8%)	11 (47.8%)
Beta-blockers	Bisoprolol, nebivolol, metoprolol, carvedilol	14 (60.9%)	16 (69.6%)
Calcium-channel blockers	Amlodipine, felodipine, lercanidipine, nicergoline*	12 (52.2%)	10 (43.5%)
Diuretics	Indapamide, furosemide, hydrochlorothiazide, spironolactone	11 (47.8%)	14 (60.9%)
Centrally acting antihypertensives	Rilmenidine, doxazosin, clonidine	6 (26.1%)	7 (30.4%)
Vasodilators / others	Nitroglycerin, trimetazidine	4 (17.4%)	6 (26.1%)
Antiplatelet / Anticoagulant agents	ASA, clopidogrel, apixaban, rivaroxaban, acenocoumarol	17 (73.9%)	19 (82.6%)
Lipid-lowering agents	Atorvastatin, rosuvastatin, fluvastatin	13 (56.5%)	18 (78.3%)
Antidiabetic therapy	Metformin, gliclazide, SGLT2 inhibitors (dapagliflozin, empagliflozin), insulin preparations	7 (30.4%)	18 (78.3%)
Thyroid hormone therapy	Levothyroxine	7 (30.4%)	5 (21.7%)
Psychotropic medications	SSRIs (escitalopram, citalopram, paroxetine), benzodiazepines (alprazolam, clonazepam), antipsychotics (quetiapine, risperidone), sedatives (zopiclone)	15 (65.2%)	17 (73.9%)
Gastroprotective agents	Proton-pump inhibitors (pantoprazole, esomeprazole, famotidine)	17 (73.9%)	19 (82.6%)
Cognitive enhancers	Donepezil, memantine, rivastigmine, piracetam	18 (78.3%)	2 (8.7%)
Respiratory medications	β_2-agonists, corticosteroid inhalers, combination inhalers	4 (17.4%)	7 (30.4%)
Anti-inflammatory / analgesic agents	NSAIDs, metamizole, diclofenac	6 (26.1%)	8 (34.8%)
Supplements / vitamins	D3, calcium, magnesium, B vitamins, ginkgo extract	21 (91.3%)	21 (91.3%)
Other medications	Diuretics, uric-acid lowering therapy (allopurinol), gastrointestinal motility agents, pancreatic enzymes, etc.	19 (82.6%)	20 (87.0%)

Medications taken by participants in the AD (n = 23) and non-AD (n = 23) groups, categorized into clinically relevant pharmacological subclasses. Values represent the number and percentage of participants receiving ≥1 medication within each class. Antihypertensives are broken down into ACE inhibitors, angiotensin II receptor blockers (ARBs), beta-blockers, calcium-channel blockers, diuretics, centrally acting agents, and vasodilators. Cognitive enhancers and psychotropic, metabolic, cardiovascular, and supportive therapies are also listed. Nicergoline is listed under calcium-channel–related vasodilators for classification purposes.

Supplementary Table 3. Routine hematological and biochemical markers in AD and non-AD groups

Category	Parameter	AD (mean ± SEM)	non-AD (mean ± SEM)	*p*-value
Hematology	White blood cells (G/L)	8.15 ± 0.91	8.70 ± 0.92	0.524
	Lymphocytes (G/L)	1.06 ± 0.25	1.00 ± 0.16	0.457
	Lymphocytes (%)	14.37 ± 2.18	12.57 ± 1.58	0.839
	Monocytes (G/L)	0.71 ± 0.05	0.66 ± 0.05	0.527
	Monocytes (%)	10.49 ± 0.94	9.43 ± 1.22	0.501
	Neutrophils (%)	74.92 ± 2.88	77.08 ± 2.61	0.581
	Eosinophils (%)	0.45 ± 0.21	0.54 ± 0.18	0.217
	Basophils (%)	0.25 ± 0.04	0.38 ± 0.06	0.077
	Red blood cells (T/L)	4.28 ± 0.14	4.45 ± 0.17	0.441
	Hemoglobin (g/L)	129.39 ± 4.46	130.35 ± 4.96	0.887
	Hematocrit (L/L)	0.379 ± 0.012	0.385 ± 0.013	0.751
	Platelets (G/L)	205.48 ± 22.45	227.04 ± 25.33	0.423
	Mean platelet volume (fL)	10.94 ± 0.21	11.22 ± 0.27	0.417
	MCHC (g/L)	340.00 ± 2.77	338.00 ± 2.87	0.618
Metabolic / biochemical	Fasting glucose (mmol/L)	6.75 ± 0.41	8.78 ± 0.79	0.086
	Albumin (g/L)	39.12 ± 1.14	40.76 ± 1.48	0.200
Liver function	LDH (U/L)	244.30 ± 20.52	269.87 ± 32.09	0.818
	GOT (U/L)	35.70 ± 8.46	81.43 ± 48.29	0.552
	GPT (U/L)	19.96 ± 2.80	49.70 ± 23.02	0.239
	**ALP (U/L)**	**78.00 ± 4.95**	**106.65 ± 11.32**	**0.025**
	**GGT (U/L)**	**31.48 ± 9.14**	**56.13 ± 21.91**	**0.006**
Kidney function & others	Sodium (mmol/L)	136.26 ± 1.26	136.22 ± 0.78	0.396
	Potassium (mmol/L)	4.19 ± 0.21	4.40 ± 0.18	0.531
	Creatinine (µmol/L)	123.00 ± 35.03	125.55 ± 17.51	0.065
	Urea (mmol/L)	9.11 ± 1.90	9.68 ± 1.10	0.135
	D-dimer (mg/L)	1.48 ± 0.47	2.31 ± 0.85	0.450
	Ferritin (ng/mL)	416.09 ± 90.36	290.38 ± 69.07	0.158
Inflammatory & cardiac	(pg/mL)	64.88 ± 11.89	113.51 ± 43.29	0.437
	(mg/L)	40.17 ± 9.51	43.65 ± 11.08	0.725
	(ng/L)	34.67 ± 6.05	33.45 ± 5.72	0.981
	CK (U/L)	213.89 ± 69.50	82.57 ± 10.38	0.419
	ProBNP (pg/mL)	1463.28 ± 482.58	2598.08 ± 627.26	0.178
	(ng/mL)	0.25 ± 0.08	0.48 ± 0.18	0.464

Data are shown as mean ± SEM. *p*-values from unpaired two-tailed *t*-tests. Parameters without significant differences are presented for transparency; significantly different values are highlighted (ALP, GGT higher in the non-AD group).

Supplementary Table 4. Tests for NfL's normal distribution in AD and non-AD groups

D'Agostino & Pearson test	non-AD	AD
K2	3,418	5,930
P value	0,1810	0,0516
Passed normality test (alpha=0.05)?	Yes	Yes
P value summary	ns	ns

Anderson-Darling test	non-AD	AD
A2*	0,6309	0,3584
P value	0,0878	0,4219
Passed normality test (alpha=0.05)?	Yes	Yes
P value summary	ns	ns

Shapiro-Wilk test	non-AD	AD
W	0,9088	0,9445
P value	0,0385	0,2243
Passed normality test (alpha=0.05)?	No	Yes
P value summary	*	ns

Kolmogorov-Smirnov test	non-AD	AD
KS distance	0,1449	0,1165
P value	>0,1000	>0,1000
Passed normality test (alpha=0.05)?	Yes	Yes
P value summary	ns	ns

Number of values	23	23

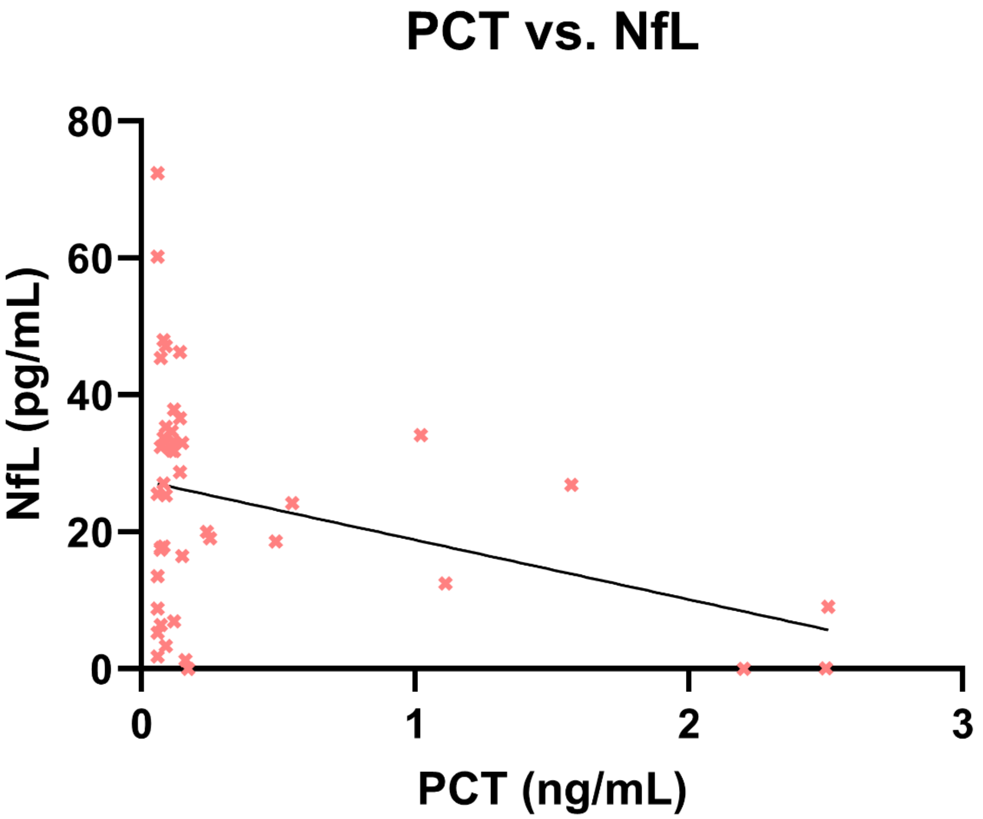

Supplementary Figure 1. Correlation between plasma NfL concentrations and procalcitonin (PCT). Scatter plot showing the relationship between plasma NfL (pg/mL) and PCT (ng/mL) levels across the full study cohort (n = 43). Each dot represents one participant. A significant negative correlation was observed (Pearson r = –0.33, 95% CI: –0.58 to –0.04; p = 0.03), suggesting that higher systemic inflammation (reflected by elevated PCT) is associated with lower circulating NfL concentrations. The solid line represents the best-fit linear regression, and the shaded area indicates the 95% confidence interval

Supplementary Table 5. Tests for the normal distribution of PBMC's SDC3 in AD and non-AD groups

D'Agostino & Pearson test	non-AD	AD
K2	1,224	2,616
P value	0,5421	0,2704
Passed normality test (alpha=0.05)?	Yes	Yes
P value summary	ns	ns

Anderson-Darling test	non-AD	AD
A2*	0,3118	0,6343
P value	0,5262	0,0860
Passed normality test (alpha=0.05)?	Yes	Yes
P value summary	ns	ns

Shapiro-Wilk test	non-AD	AD
W	0,9742	0,9308
P value	0,7892	0,1140
Passed normality test (alpha=0.05)?	Yes	Yes
P value summary	ns	ns

Kolmogorov-Smirnov test	non-AD	AD
KS distance	0,1161	0,1774
P value	>0,1000	0,0587
Passed normality test (alpha=0.05)?	Yes	Yes
P value summary	ns	ns

Number of values	23	23

Supplementary Table 6. Correlations of PBMC-expressed SDC3 with systemic laboratory markers.

Parameter	Correlation coefficient	95% CI	p-value
PCT	r = –0.439	–0.654 to –0.160	0.0032
ALP	r = –0.416	–0.630 to –0.143	0.0040
CRP	r = –0.331	–0.572 to –0.037	0.0283
Urea	ϱ = –0.323	–0.567 to –0.027	0.0285
NfL	r = 0.244	–0.049 to 0.509	0.102

Correlation coefficients (Pearson or Spearman), 95% confidence intervals, and p-values are listed for each parameter. PBMC-expressed SDC3 showed significant inverse associations with inflammatory and metabolic markers (PCT, ALP, CRP, urea), while no significant correlation was observed with plasma NfL.

Supplementary Table 7. Tests for the normal distribution of plasma SDC3 in AD and non-AD groups

D'Agostino & Pearson test	non-AD	AD
K2	5.439	3.963
P value	0.0659	0.1379
Passed normality test (alpha=0.05)?	Yes	Yes
P value summary	ns	ns

Anderson-Darling test	non-AD	AD
A2*	0.3149	0.7053
P value	0.5203	0.0566
Passed normality test (alpha=0.05)?	Yes	Yes
P value summary	ns	ns

Shapiro-Wilk test	non-AD	AD
W	0.9577	0.9122
P value	0.4176	0.0455
Passed normality test (alpha=0.05)?	Yes	No
P value summary	ns	*

Kolmogorov-Smirnov test	non-AD	AD
KS distance	0.1235	0.1552
P value	>0,1000	>0,1000
Passed normality test (alpha=0.05)?	Yes	Yes
P value summary	ns	ns

Number of values	23	23

Supplementary Table 8. Correlations of plasma SDC3 with systemic laboratory parameters.

Parameter	r (Pearson/Spearman)	95% CI	*p*-value
ALP	–0.425	–0.630 to –0.143	0.0032
PCT	–0.392	–0.617 to –0.115	0.0094
CRP	–0.375	–0.604 to –0.098	0.0103
NfL	0.244	–0.050 to 0.508	0.102

Pearson correlation coefficients (r), 95% confidence intervals (CI), and p-values are shown.
Plasma SDC3 levels were inversely associated with inflammatory and hepatic markers (ALP, PCT, CRP), while no significant association was observed with neurofilament light chain (NfL).

Supplementary Table 9. Technical replicate consistency of plasma NfL ELISA measurements

Sample	Replicate 1 (pg/mL)	Replicate 2 (pg/mL)	Mean (pg/mL)	% Difference*
1	29.36	34.47	31.92	16.0
2	22.59	25.88	24.23	13.6
3	44.64	51.37	48.00	14.0
4	41.39	49.53	45.46	17.9
5	29.77	33.86	31.82	12.9
6	35.89	39.96	37.93	10.7
7	17.82	20.31	19.07	13.1
8	29.98	34.90	32.44	15.2
9	32.82	37.95	35.38	14.5
10	8.16	9.43	8.80	14.4
11	68.93	75.92	72.42	9.6
12	11.76	13.23	12.49	11.8
13	25.06	29.16	27.11	15.1
14	33.24	36.10	34.67	8.3
15	15.73	19.08	17.41	19.2
16	17.05	20.18	18.62	16.8
17	24.65	29.16	26.90	16.8
18	49.73	45.45	47.59	9.0
19	34.67	38.54	36.61	10.6
20	24.03	27.11	25.57	12.0
21	35.89	31.61	33.75	12.7
22	16.37	19.49	17.93	17.4
23	49.53	43.01	46.27	14.1
24	0.08	0.14	0.11	54.5†
25	1.38	1.22	1.30	12.3
26	1.73	1.95	1.84	12.0
27	3.03	3.67	3.35	19.1
28	6.24	6.67	6.46	6.7
29	6.46	7.52	6.99	15.2
30	12.81	14.28	13.54	10.9
31	8.59	9.65	9.12	11.6
32	16.16	19.28	17.72	17.6
33	15.11	17.82	16.47	16.5
34	18.02	21.98	20.00	19.8

Sample	Replicate 1 (pg/mL)	Replicate 2 (pg/mL)	Mean (pg/mL)	% Difference*
35	4.95	5.62	5.28	12.7
36	25.47	29.77	27.62	15.6
37	24.03	26.70	25.37	10.5
38	25.06	29.77	27.42	17.2
39	26.29	31.20	28.75	17.1
40	34.47	31.61	33.04	8.7
41	30.18	36.10	33.14	17.9
42	31.00	37.32	34.16	18.5
43	43.42	50.96	47.19	16.0
44	54.64	65.79	60.22	18.5
45	0.00	0.00	0.00	0‡
46	0.00	0.00	0.00	0‡

* % difference was calculated as: (Replicate 1 − Replicate 2) / mean × 100.

† Elevated percentage difference reflects very low absolute concentrations near the assay detection limit.

‡ Zero signal in both technical replicates.
